# Fauna and Geographical Distribution of Scorpions in Ilam Province, South Western Iran

**Published:** 2017-05-27

**Authors:** Narges Sharifinia, Iman Gowhari, Manijeh Hoseiny-Rad, Ali Ashraf Aivazi

**Affiliations:** 1 Department of Public Health, School of Health, Ilam University of Medical Sciences, Ilam, Iran; 2 Department of Biology, Payam-e-Noor University, Ilam Branch, Ilam, Iran; 3 Department of Biology, Farhangian University, Tehran, Iran

**Keywords:** Habitat, Scorpion, Fauna, Ecology, Iran

## Abstract

**Background:**

Scorpions’ stings and their own mortalities place them among the most important health and medical problems. The dreadful features and especially their poisonous stings are considered a major cause of human stress and abhorrence/phobia. The current study aimed to study the scorpion fauna of Ilam Province, south western Iran in order to manage scorpionism related problems.

**Methods:**

In this field-laboratory investigation during March 2014 to February 2015, different parts of Ilam Province were surveyed. Nine sampling parts were selected based on geographical situation, scorpionism reports, weather, flora, and local data. Capturing scorpion was done employing a black light, and a long forceps from dusk to midnight. The collected scorpions were placed to 70% ethyl alcohol. All specimens were determined based on the valid taxonomic keys, furthermore their sexes were studied.

**Results:**

Out of the 391 collected scorpions, 11 species were identified as follows: *Hottentotta saulcyi*, *Mesobuthus eupeus*, *Compsobuthus matthiesseni*, *Razianus zarudnyi*, *Hemiscorpius lepturus*, *Androctonus crassicauda*, *Orthochirus iranus*, *Odontobuthus bidentatus*, *Buthacus macrocentrus*, *Scorpio maurus*, and *Polisius persicus*.

**Conclusion:**

Eleven species of Buthidae, Scorpionidae and Hemiscorpiidae families from high risk areas were identified. Despite the low surface of the province, such different species reveals a diverse scorpion fauna that, in turn, shows good and suitable habits of scorpions, as considered by health staff.

## Introduction

The fearful feature and painful poisonous stings of scorpions have caused human phobia for a long time. Most people think of scorpions as pests and killers of man (Polis 1990). Scorpions having diverse distribution are mostly living in semi-temperate regions at latitude of 23–38 °C, while their abundance and diversity toward Equator and also poles decrease (Polis 1990). Out of about 1,500 scorpion species in the world, few cause severe toxicity, including more than 1.23 million stings annually, of which approximately 3,250 (0.27%) cause death (Khatony et al. 2015). Mexico, Colombia, and Iran are the most affected countries.

Out of about 100,000 scorpionism cases including children (75%) in Iran, only 36,000–50,000 ones are reported officially, with a 7–60 mortality rate per year (Ghaderi 2004, Zarei et al. 2009, Mirshamsi et al. 2011), especially in Khuzestan, Bushehr and Ilam Provinces (south-western Iran) (Rafizadeh et al. 2013).

The Iranian scorpion fauna ranges from 51–66 species in 17–23 genera and 3–4 families, according to different references (Mirshamsi et al. 2011, Navidpour 2012), of which about 10 species have been incriminated in human envenomation, that, in turn, is more than any other country in Middle East (Dehghani and Fathi 2012). Except *Hemiscorpius lepturus* (Hemiscorpiidae), the most important, medically, scorpion in Iran (Kovařík 1997), all the venomous scorpion species belong to the Buthidae family (Zarei et al. 2009).

Ilam Province (Latitude: 33° 63′ 74″ N, Longitude: 46° 42′ 27″ E) located at west of Iran with around 20,150km^2^ and 1.2% surface of the country area, rich and diverse plant coverage, and also various climates, is of the most suitable living-places for scorpions. The current study aimed at determining fauna and bio-geographical distribution of scorpions in Ilam in 2014–15.

## Materials and Methods

**Fig. 1.**
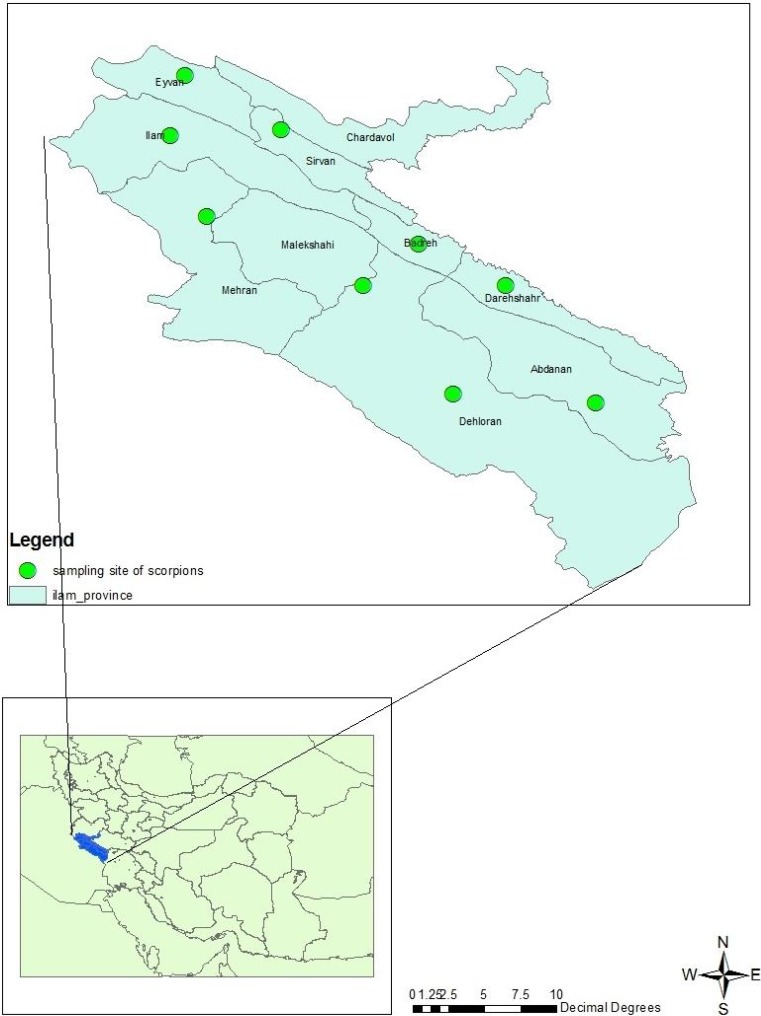
Sampling sites of scorpions in Ilam Province, 2014

In this cross-sectional study of employing field and laboratory techniques, the fauna was investigated during March 2014 to February 2015. The sampling sites (nine places) were selected based on geographical situation, scorpionism reports, weather, and plant coverage in all the three climates of the province. The detailed data of sampling sites have been shown in Table 1 and Fig. 1.

**Table 1.** Sampling sites of scorpions in Ilam Province, 2014

**Climate type**	**County**	**Sampling site**	**Longitude**	**Altitude**	**Height (sea level m)**
**Cold mountainous**	Aivan	Babagir	46◌ْ11◌َ	33◌ْ56◌َ	1,070
**Cold mountainous**	Ilam	Gholandar	46◌ْ27◌َ	33◌ْ39◌َ	1,045
**Cold mountainous**	Sirvan	Karezan	46◌ْ32◌َ	33◌ْ44◌َ	1,280
**Moderate mountainous**	Badre	Badre	47◌ْ1◌َ	33◌ْ19◌َ	1,090
**Moderate mountainous**	Dare-shahr	Dare-shahr	47◌ْ21◌َ	33◌ْ10◌َ	670
**Moderate mountainous**	Zarin-abad	Sayed-naseredin village	46◌ْ50◌َ	33◌ْ10◌َ	810
**Dry and hot**	Abdanan	Murmuri	47◌ْ41◌َ	32◌ْ44◌َ	520
**Dry and hot**	Mehran	Golan	46◌ْ16◌َ	33◌ْ25◌َ	550
**Dry and hot**	Dehloran	Bishe-deraz	47◌ْ01◌َ	32◌ْ46◌َ	390

The sampling was done at night (from dusk to midnight) using a black light -ultra violet (UV) light- and a long forceps. All the captured scorpions were placed in 70% ethyl alcohol. No specimens were caught by abovementioned method during November to February months, due to cold weather of sampling areas. The geographical data were recorded by a GPS apparatus (GARMIN 78 S).

All the specimens were identified according to taxonomic keys (Navidpour et al. 2008a). The gender of specimens was also determined based on Farzanpey method (Farzanpay and Vachon 1979).

## Results

Out of all the 391 specimens collected from nine sites in different parts of the province, 11 species were identified as follows: *Hottentotta saulcyi*, *Mesobuthus eupeus*, *Compsobuthus matthiesseni*, *Razianus zarudnyi*, *Hemiscorpius lepturus*, *Androctonus crassicauda*, *Orthochirus iranus*, *Odontobuthus bidentatus*, *Buthacus macrocentrus*, *Scorpio maurus*, and *Polisius persicus.*

The most abundant species were *H. saulcyi* 25.09% (relative frequency), *M. eupeus* 23.29%, and *C. matthiesseni* 16.18% which showed the highest frequency in all the three climates studied. The lowest abundance stood for *P. persicus* 1.79% and *S. maurus* 1.29%, respectively. *H. lepturus* which is the most poisonous scorpion of Iran had a 7.16% relative frequency. Further, *R. zarudnyi* was found just in mild mountainous climate, however, *O. bidentatus* 3.58% and *B. macrocentrus* 2.84% were found in low abundance at dryhot climate. Totally, 138 males and 253 females were identified showing F/M sex ratio of about 2:1 (Table 2).

**Table 2.** Scorpions collected in different parts of Ilam Province, 2014

**Family**	**species**	**Sampling sites**	**Frequency of each species**			**Relative frequency at the province (%)**
			**Male N (%)**	**Female N (%)**	**Total**	
**Buthidae**	*Hottentotta saulcyi*	All areas	30 (31)	68 (69)	98	25.09
**Buthidae**	*Mesobuthus eupeus*	All areas	40 (43)	52 (57)	92	23.29
**Buthidae**	*Compsobuthus matthiesseni*	Ilam, Sirvan, Dareh-shahr, Zarin-abad	22 (35)	41 (65)	63	16.18
**Buthidae**	*Razianus zarudnyi*	Mehran, Badre, Zarin-abad	16 (47)	18 (53)	34	8.69
**Hemiscorpiidae**	*Hemiscorpius lepturus*	Mehran, Badre, Abdanan	10 (36)	18 (64)	28	7.19
**Buthidae**	*Androctonus crassicauda*	Sirvan, Mehran, Dehloran, Ilam, Aivan	5 (24)	16 (76)	21	5.39
**Buthidae**	*Orthochirus iranus*	Sirvan, Mehran, Dehloran, Ilam, Aivan	6 (33)	12 (67)	18	4.67
**Buthidae**	*Odontobuthus bidentatus*	Dehloran, Abdanan	2 (14)	12 (86)	14	3.58
**Buthidae**	*Buthacus macrocentrus*	Dehloran, Abdanan	3 (27)	8 (73)	11	2.84
**Buthidae**	*Polisius persicus*	Abdanan	3 (43)	4 (57)	7	1.79
**Scorpionidae**	*Scorpio maurus*	Dehloran, Abdanan	1 (20)	4 (80)	5	1.29
**All Scorpion species**			138 (35.30)	253 (64.70)	391	100

## Discussion

In the current study, totally 11 scorpion species from Buthidae, Scorpionidae, and Hemiscorpiidae families were identified, which shows a diverse fauna due to the good habitat and favorite climate of the studied area. In other studies accomplished in Iran, 8 species from Fars and Kohgilouyeh and Boyer-Ahmad provinces (Azizi et al. 2001), 10 species from Hormozgan Province (Shahi et al. 2009), 3 species from Gonabad County (Ramezani-Avval-Riabi et al. 2010), 8 species from Kerman Province (Dehghani 2008), 7 species from Kish Island (Khaghani et al. 2005), 7 species from Qeshm Island (Zarei et al. 2009), 8 species from Sistan and Balouchestan Province (Nejati et al. 2014), 2 species from Sari County (Motavali-Haghi et al. 2004), 5 species from Chaharmahal and Bakhtiari Province (Pirali-Kheirabadi et al. 2014), and finally 5 species from Zanjan Province (Moradi et al. 2015) have been reported. Comparing the current findings to other studies, the species richness and diversity of scorpions can be concluded, based on the quantity of species found. However, in neighboring provinces of Lorestan, and Khuzestan with almost similar climates, 5 and 19 species have been reported respectively, showing much more diversity in Khuzestan Province (Taherian 2003, Navidpour et al. 2008b).

In the current study, 11 species including *H. saulcyi*, *M. eupeus*, *C. matthiesseni*, *R. zarudnyi*, *H. lepturus*, *A. crassicauda*, *O. iranus*, *O. bidentatus*, *B. macrocentrus*, *S. maurus*, and *P. persicus* were collected and identified in Ilam Province. Mozafari had reported 7 species in one county of the province (Mozaffari et al. 2013), Gowhari had identified 10 species in different climates of the province (Gowhari et al. 2012), however, Navidpour reported 14 species in three families, while *Vachoniolus iranus*, *Compsobuthus jakesi*, *Apistobuthus susanae* species were not found in our study. The recent species had been found in Ein-e-Kosh village (Navidpour et al. 2008a), located in far south of the province, having a similar climate and ecosystem to that of Khuzestan Province. Therefore, no sampling has been done in that area, a fact that justifies the difference.

The most abundant species of the province was *H. saulcyi* which collected in all the three climatic areas of the province, as well reported by Gowhari in all studied places (Gowhari et al. 2012). Sedaghat (Sedaghat et al. 2012), in respect to biogeographical distribution of Iran’s scorpions has reported the *H. saulcyi* in Khuzestan, Kohgiloyeh-Boirahmad, and Kermanshah Provinces with dryhot, cold-mountainous, and mild-mountainous climates, respectively, showing high adaptation of the species.

The second abundant species was *M. eupeus* found in mountainous areas and beneath the rocks, that were in accordance with the findings of Khairabadi in Chaharmahal and Bakhtiari mountainous areas, and also those of Motavali-Haghi from mountains of Sari County, northern Iran (Motavali-Haghi et al. 2004, Pirali-Kheirabadi et al. 2014).

Out of the most dangerous and venomous scorpions, *H. lepturus* and *A. crassicauda* (black scorpion) with frequencies of 7% and 5%, respectively, were also found in the studied area. The *H. lepturus* has been reported as the most dangerous and main cause of death in Khuzestan Provinces’ scorpionism (Radmanesh 1990, Dehghani and Fathi 2012, Nejati et al. 2014). The thin and small sting has been reported as the feature of such species, along with a painless sting which leads to acute complications such as tissue necrosis, hemolysis, and even death during the first 48 hours. *Hemiscorpius lepturus* was identified in Golan, Badre, and Murmuri areas of the province.

*Androctonus crassicauda* was found in five areas of the province (Table 2) reported as the main scorpionism cause in Khuzestan Province. The current species have also been reported in Semnan, Bushehr, and Lorestan provinces (Sedaghat et al. 2012).

From the sex-ratio point of view, the females were the dominant gender during the study, i.e. 2:1 (F/M) sex ratio. Wilson has reported the sex-ratio (F/M) of 3.91 and/or 4:1 (Lourenço 2002). Shahi has also reported much more abundance of females than males in Hormozgan Province which both are in accordance with our findings (Shahi et al. 2009).

## Conclusion

Despite the relatively small area (1.2% of the country surface), a diverse fauna was seen, compared to other studies in different provinces of Iran. Such geographical distribution may be affected by climate changes and global warming, their habitat, and even the distribution pattern of each species (Bellard et al. 2012). The health and medical importance of scorpions necessitates comprehensive and periodic research on their ecology including habitat, diet, environment’s temperature, humidity, and precipitation in the province.

## References

[B1] Azizi K Shahraki G Omrani M (2001) Determining the scorpion fauna of homes and farms in the surrounding villages of Kohgiluyeh and Boyer-Ahmad, 2000. Armaghane Danesh. 22: 6–13 (in Persian).

[B2] Bellard C Bertelsmeier C Leadley P Thuiller W Courchamp F (2012) Impacts of climate change on the future of biodiversity. Ecol Lett. 15: 365–377. 22257223 10.1111/j.1461-0248.2011.01736.x PMC3880584

[B3] Dehghani R Fathi B (2012) Scorpion sting in Iran: a review. Toxicon. 60: 919–933. 22750221 10.1016/j.toxicon.2012.06.002

[B4] Dehghani R Moabed S Kamyabi F Haghdoost AA Mashayekhi M Soltani H (2008) Scorpions fauna of Kerman Province-Iran. J Kerman Uni Med Sci. 15: 172–181 (in Persian).

[B5] Farzanpay R Vachon M (1979) Contribution à l’étude des caractères sexuels secondaires chez les scorpions Buthidae (Arachnida). Revue Arachnologique. 2: 137–142 (in French).

[B6] Ghaderi H (2004) Evaluation of scorpion bites in the military soldiers in North-Western part of Khuzestan Province from May 2002 to December 2003. J Mil Med Sci Iran. 2: 451–455 (in Persian).

[B7] Gowhari I Pashaeirad S Navidpour S Molaei-Birgani S (2012) Study on scorpion fauna of Ilam province, western of Iran. J Exp Anim Biol. 1: 43–47 (in Persian).

[B8] Khaghani R Tirgari S Omrani G Rafinejad J Mousavi-Eyvanaki A (2005) Faunestic study and distribution of scorpions in Kish Island, Iran (Persian Gulf). Modares J Med Sci. 8: 7–11 (in Persian).

[B9] Khatony A Abdi A Fatahpour T Towhidi F (2015) The epidemiology of scorpion stings in tropical areas of Kermanshah Province, Iran, during 2008–2009. J Venom Anim Toxins Incl Trop Dis. 21: 45. 26550009 10.1186/s40409-015-0045-4 PMC4636075

[B10] Kovařík F (1997) Results of the Czech Biological Expedition to Iran. Part 2. Arachnida: Scorpiones, with descriptions of *Iranobuthus krali* gen. n. et sp. n. and *Hottentotta zagrosensis* sp. n. (Buthidae). Acta Soc Zool Bohem. 61: 39–52.

[B11] Lourenco WR (2002) Reproduction in scorpions, with special reference to parthenogenesis. European Arachnology 2000: Proceedings of the 19th Colloquium of Arachnology, Aarhus: Aarhus University Press.

[B12] Mirshamsi O Sari A Hosseinie S (2011) History of study and checklist of the scorpion fauna (Arachnida: Scorpiones) of Iran. Progress Biol Sci. 1: 16–23.

[B13] Moradi M Yagmur E Pooyan-Moradi G Ahmadi F (2015) Scorpion fauna of Zanjan Province, Iran (Arachnida: Scorpiones). J Appl Biol Sci. 9: 11–14.

[B14] Motavali-Haghi F Tirgari S Çhangani F Mohammadpour RA (2004) A study on the scorpin species of the mountainous areas of Sari township in 2001. J Mazandaran Univ Med Sci. 14: 92–96 (in Persian).

[B15] Mozaffari E Sedaghat M Sanei-Dehkordi A Akbarzadeh K (2013) Biodiversity and species composition of scorpions (Arachnida, Scorpiones) in Ilam County, Iran. J Appl Sci Res. 9: 5412–5418.

[B16] Navidpour S (2012) Scorpions of Iran. Karaj, Iran: Razi Vaccine and Serum Research Institute [Accessed 06/12/2015], Available at: http://www.scorpion-research.ir/.

[B17] Navidpour S Fet V Kovařík F Soleglad M (2008a) Scorpions of Iran (Arachnida, Scorpiones). Part III. Ilam Province. Euscorpius. 69: 1–29.

[B18] Navidpour S Kovařík F Soleglad M Fet V (2008b) Scorpions of Iran (Arachnida, Scorpiones). Part IV. Kohgilouyeh and Boyer Ahmad Province. Euscorpius. 74: 1–24.

[B19] Nejati J Mozafari E Saghafipour A Kiyani M (2014) Scorpion fauna and epidemiological aspects of scorpionism in southeastern Iran. Asian Pac J Trop Biomed. 4: 217–221. 10.12980/APJTB.4.2014C1323 PMC4025348 25183084

[B20] Pirali-Kheirabadi K Khalaji-Pirbalouty V Jazayeri A (2014) Geographical distribution of scorpions in Chaharmahal and Bakhteyari Province. Veterinary J. 27: 45–51 (in Persian).

[B21] Polis GA (1990) The biology of scorpions. Stanford University Press, Stanford.

[B22] Radmanesh M (1990) Clinical study of *Hemiscorpius lepturus* in Iran. J Trop Med Hyg. 93: 327–332. 2231841

[B23] Rafizadeh S Rafinejad J Rassi Y (2013) Epidemiology of scorpionism in Iran during 2009. J Arthropod Borne Dis. 7: 66–70. 23785696 PMC3684498

[B24] Ramezani-Avval-Riabi H Matlabi M Rafinejad J Amiri M (2010) The ecofaunistics of scorpions in Gonabad. Horizon Med Sci. 15: 54–61 (in Persian).

[B25] Sedaghat M Salehi-Moghadam A Dehghani R (2012) Mapping the distribution of some important scorpions collected in the past five decades in Iran. J Mil Med Sci Iran. 9: 285–296 (in Persian).

[B26] Shahi M Azizi K Ansarian N (2009) Study on scorpions fauna in high risk area of Hormozgan Province, 2006–2007. Hormozgan Med J. 12: 207–214 (in Persian).

[B27] Taherian M (2003) Identifying scorpion fauna of scorpion of Khorramabad County. J Luristam Univ Med Sci. 5: 43–45 (in Persian).

[B28] Zarei A Rafinejad J Shemshad K Khaghani R (2009) Faunistic study and biodiversity of scorpions in Qeshm Island (Persian Gulf). Iran J Arthropod Borne Dis. 3: 46–52. 22808372 PMC3385527

